# Performance of Risk Models for Antimicrobial Resistance in Adult Patients With Sepsis

**DOI:** 10.1001/jamanetworkopen.2024.43658

**Published:** 2024-11-07

**Authors:** M. Cristina Vazquez Guillamet, Hanyang Liu, Andrew Atkinson, Victoria J. Fraser, Chenyang Lu, Marin H. Kollef

**Affiliations:** 1 Division of Infectious Diseases, Department of Medicine, Washington University School of Medicine, St Louis, Missouri; 2 Division of Pulmonary and Critical Care Medicine, Department of Medicine, Washington University School of Medicine, St Louis, Missouri; 3 McKelvey School of Engineering, Department of Computer Science & Engineering, Washington University, St Louis, Missouri; 4 Department of Medicine, Washington University School of Medicine, St Louis, Missouri

## Abstract

**Importance:**

The results of prediction models that stratify patients with sepsis and risk of resistant gram-negative bacilli (GNB) infections inform treatment guidelines. However, these models do not extrapolate well across hospitals.

**Objective:**

To assess whether patient case mix and local prevalence rates of resistance contributed to the variable performance of a general risk stratification GNB sepsis model for community-onset and hospital-onset sepsis across hospitals.

**Design, Setting, and Participants:**

This was a retrospective cohort study conducted from January 2016 and October 2021. Adult patients with sepsis at 10 acute-care hospitals in rural and urban areas across Missouri and Illinois were included. Inclusion criteria were blood cultures indicating sepsis, having received 4 days of antibiotic treatment, and having organ dysfunction (vasopressor use, mechanical ventilation, increased creatinine or bilirubin levels, and thrombocytopenia). Analyses were completed in April 2024.

**Exposure:**

The model included demographic characteristics, comorbidities, vital signs, laboratory values, procedures, and medications administered.

**Main Outcomes and Measures:**

Culture results were stratified for ceftriaxone-susceptible GNB (SS), ceftriaxone-resistant but cefepime-susceptible GNB (RS), and ceftriaxone- and cefepime-resistant GNB (RR). Negative cultures and other pathogens were labeled SS. Deep learning models were developed separately for community-onset (patient presented with sepsis) and hospital-onset (sepsis developed ≥48 hours after admission) sepsis. The models were tested across hospitals and patient subgroups. Models were assessed using area under the receiver operating characteristic curve (AUROC) and area under precision recall curve (AUPRC).

**Results:**

A total of 39 893 patients with 85 238 sepsis episodes (43 207 [50.7%] community onset; 42 031 [48.3%] hospital onset) were included. Median (IQR) age was 65 (54-74) years, 21 241 patients (53.2%) were male, and 18 830 (47.2%) had a previous episode of sepsis. RS contributed to 3.9% (1667 episodes) and 5.7% (2389 episodes) of community-onset and hospital-onset sepsis episodes, respectively, and RR contributed to 1.8% (796 episodes) and 3.9% (1626 episodes), respectively. Previous infections and exposure to antibiotics were associated with the risk of resistant GNB. For example, in community-onset sepsis, 375 RR episodes (47.1%), 420 RS episodes (25.2%) and 3483 of 40 744 (8.5%) SS episodes were among patients with resistance to antimicrobial drugs (P < .001). The AUROC and AUPRC results varied across hospitals and patient subgroups for both community-onset and hospital-onset sepsis. AUPRC values correlated with the prevalence rates of resistant GNB (*R* = 0.79; *P* = .001).

**Conclusions and Relevance:**

In this cohort study of 39 893 patients with sepsis, variable model performance was associated with prevalence rates of antimicrobial resistance rather than patient case mix. This variability suggests caution is needed when using generalized models for predicting resistant GNB etiologies in sepsis.

## Key Points

**Question:**

Do patient case mix and local resistance rates affect the performance of antimicrobial resistance risk stratification models in community-onset and hospital-onset sepsis?

**Findings:**

This retrospective cohort study of 39 893 adult patients with sepsis included 4.8% infections caused by ceftriaxone-resistant gram-negative bacilli (GNB) and 2.8% caused by cefepime-resistant GNB. Risk stratification model performance correlated with the prevalence of resistance rates and varied widely across participating hospitals and patient subgroups.

**Meaning:**

The variable model performance observed in this study argues against the use of generalized models in predicting resistant GNB etiologies in sepsis.

## Teaser

This cohort study evaluates the performance of a risk stratification model for resistant gram-negative bacilli infection among patients with sepsis.

## Introduction

Empirical antibiotics that match in vitro susceptibility are crucial in the treatment of serious infections.^1^ The underuse and overuse of broad-spectrum antibiotics is associated with detrimental outcomes, including increased mortality.^2,3,4,5^ Prediction models have been developed to stratify patients at risk for infections caused by resistant gram-negative bacilli (GNB) and to help clinicians decide between narrow- or broad-spectrum empirical antibiotics. These models had promising performance in the derivation population, but very few were validated in different cohorts.^6^ Current sepsis guidelines rely on these models to define comorbidities associated with higher risk of resistant GNB across hospitals while also emphasizing the importance of local resistance rates.^1^ We hypothesized that differing patient case mixes would explain the variable performance of resistant GNB prediction models across hospitals. Case mix indexes represent the average diagnosis relative weight for each hospital. For this study, we deconstructed case mix into patient subgroups of interest. In other words, we hypothesized that, eg, patients with leukemia would carry a similar risk of resistant infections across hospitals, but their proportion within the hospitals’ patient population would differ. Additionally, we hypothesized that local GNB resistance rates affected models’ performance. We tested our hypotheses by developing deep learning models based on artificial neural networks to model complex medical data and to project levels of antimicrobial resistance in GNB responsible for community-onset and hospital-onset sepsis. We assessed model performance across patient subgroups that contribute to the case mix. Generalizable prediction models would facilitate antibiotic standardization and monitoring.

## Methods

### Study Population and Data Source

This retrospective cohort study was performed at Washington University and conducted according to the Washington University School of Medicine Institutional Review Board protocol, which approved the study and granted a waiver of consent due to the retrospective nature of the study. We report our results according to the Strengthening the Reporting of Observational Studies in Epidemiology (STROBE) reporting guideline^7^ and published recommendations for machine learning models.^8,9^ Adult patients who met the US Centers for Disease Control and Prevention (CDC) adult sepsis event criteria admitted to any of the 10 acute-care BJC hospitals (located across the greater St Louis region, including urban and rural areas of Missouri and Illinois) between January 2016 to October 2021 were eligible for inclusion. Inclusion criteria combined presumed infection (blood cultures and ≥4 qualifying days of antibiotics) with concomitant organ dysfunction (any of vasopressor use, mechanical ventilation, increased creatinine or bilirubin levels, and thrombocytopenia).^10^ Data were directly extracted and timestamped as recorded in the electronic health record (EHR).

### Outcomes

The outcome of interest was GNB antimicrobial resistance level in community-onset and hospital-onset sepsis episodes categorized to replicate choices made in clinical practice: susceptible to ceftriaxone (SS), resistant to ceftriaxone and susceptible to cefepime (RS), and resistant to both ceftriaxone and cefepime (RR). Negative cultures and cultures with non-GNB organisms were labeled SS because treatment would not require a broad(er) anti-GNB antibiotic (eg, methicillin-resistant *Staphylococcus aureus*, viruses). The most resistant GNB microbe determined the category for polymicrobial cultures. We used in vitro antimicrobial susceptibility results documented in the EHR to label sepsis episodes. BJC hospitals use Clinical and Laboratory Standards Institute guidelines to assign susceptible, intermediate, and resistant isolates based on minimal inhibitory concentration.^11^ Intermediate isolates were labeled as susceptible. If antimicrobial susceptibility results were missing from the EHR, general logic rules for intrinsic resistance were applied (eg, *Pseudomonas aeruginosa* isolates were considered resistant to ceftriaxone). Risk was stratified at the sepsis-episode level, and hospital-onset infections were separated from community-onset infections if the culture collection timestamp was more than 48 hours after admission. We defined prevalence rates for the entire study period as the proportion of episodes caused by RS and RR isolates relative to the total number of sepsis episodes.

### Statistical Analysis

#### Feature Selection, Engineering, and Data Processing

The full list of features and preprocessing details is presented in the eTable in Supplement 1. We included demographic characteristics (age, sex, race as self-reported by the patients and recorded in the EHR), *International Statistical Classification of Diseases and Related Health Problems, Tenth Revision *(*ICD-10*) codes, hospital admission history, vital signs, laboratory results, procedures, medications administered, and culture data with antimicrobial susceptibility results. Patient verbalized their race. All responses except for African American or Black and White were grouped into a single category. We selected our study period after the transition to *ICD-10* codes and mapped previous *International Classification of Diseases, Ninth Revision *(*ICD-9*) codes to current *ICD-10* codes.^12^ Prediction of antimicrobial resistance must occur at suspicion of infection to initiate empirical antibiotics. Thus, all input variables were screened at the culture timestamp. We developed 2 machine learning models (details provided in the next section), without any preprocessing such as grouping and exclusion for comorbidities. All vital signs and laboratory data points within 5 days of the culture timestamp were resampled, and all the mean values of every 4-hour window were used by the deep learning model. We defined plausible ranges for the vital signs and laboratory data; implausible values were excluded. Shock was defined as vasopressor or inotrope use within 24 hours of culture timestamps. Age, time since admission, vital signs, and laboratory results were treated as continuous variables; hospital, sex, and race were treated as categorical variables. Procedures, antibiotic administration, comorbidities, and previous susceptible or resistant culture results were treated as binary.

Laboratory value and vital sign features were selected considering redundancy, collinearity, and availability in the EHR (eg, heart rate, pulse, pulse on arterial line). For hospital-onset sepsis, we added time since admission, systemic antibiotics administered, and results from previous cultures during the same admission. We removed patients with any missing categorical feature (eg, sex and race) and carried forward the last observation for missing laboratory results and vitals. Continuous features are reported as medians with IQRs or numbers with percentages if categorical or binary. *P* < .05 was considered significant, and tests for *P* were 2-tailed. Analyses were conducted with PyTorch version 2.1 and Python version 3.8.8 (Python Software Foundation).

#### Training and Testing Machine Learning Models and Statistical Analyses

We developed 2 machine learning models with the deep learning model as the main tool. A gradient-boosting model was dedicated to variable importance (Shapley additive explanations [SHAP]) for the first 10 features contributing to the model.^13^ A positive SHAP value indicates that the feature was associated with increased risk for the studied outcome, whereas a negative SHAP value indicates that the feature was associated with lowered risk. The SHAP value magnitude reflects the feature’s contribution to the model’s performance. Gradient boosting builds decision trees sequentially by randomly sampling a subset of patients and features at each iteration while learning from past errors.^14^

The deep learning model stratified patients according to their risk for sepsis with resistant GNB categories SS, RS, and RR (as described earlier in the Methods section). The model relied on a specifically designed deep neural network architecture that processed different types of patient data using a different subnetwork for patient representation learning. Static (tabular) data (eg, demographic characteristics, hospitalization history, and vasopressor use) were processed by fully connected layers. Longitudinal data (eg, vital signs and laboratory results) were processed by recurrent neural networks to capture temporality. All comorbidities were processed by a transformer^15^ that initialized the representation of each *ICD* code using a pretraining process^16^ to preserve the intrinsic hierarchical structure of the *ICD-10* codes. The representations from each subnetwork were combined and used for antimicrobial resistance prediction.

We split the data as follows: 80% to train the models (70% for training and 10% for model selection) and 20% as a holdout set for patient group–specific testing (ie, infections from the same patient only exist in either the training or testing set). Models were trained on the whole training set and tested separately for community-onset and hospital-onset sepsis. We provide the results from the holdout testing dataset without oversampling. The RR and RS isolates had low prevalence rates; therefore, we oversampled these minority groups using the synthetic minority oversampling technique^17^ and reweighed loss of the minority group using focal loss^18^ without changes in model performance.

Model performance was evaluated by computing the area under the receiver operating characteristic curve (AUROC) and the area under the precision recall curve (AUPRC). To assess the association of patient case mix with model performance, we predefined the subgroup analyses based on severity of presentation (shock and mechanical ventilation), demographic characteristics (age <65 and ≥65 years), and comorbidities associated with antimicrobial resistance in GNB (eg, hematological malignant neoplasms, cirrhosis, previous infections with resistant GNB, and history of antibiotic exposure). The gradient-boosting model contributed to defining the subgroups of interest. We provide the deep learning model performance in 3 hospitals chosen for size and patient comorbidities. Hospital 1 is an academic quaternary center delivering care to a socioeconomically diverse population. It is a transplant and clinical trial referral center. Hospital 2 is a suburban community hospital that provides health care services to an affluent population. Hospital 3 is community-based and serves a socioeconomically disadvantaged population.

The gradient-boosting model was implemented using XGBoost version 2.0.0 (XGBoost Contributors), with tuned tree depth and feature subsampling. The machine learning pipeline of training and evaluation was implemented on scikit-learn version 1.3.0 and Python version 3.8.8. The deep learning model was implemented on PyTorch version 2.1 and Python version 3.8.8 with tuned network width and depth and learning rate. A 10% random validation split of the training set was used for both models to monitor validation performance during training and terminate training before overfitting.

## Results

A total of 40 118 patients met the CDC adult sepsis event criteria and were admitted to any of the 10 acute care BJC hospitals between January 2016 and October 2021. We excluded 148 patients due to missing antimicrobial susceptibility results and 77 due to missing sex or race. The median (IQR) age was 65 (54-74) years, and 21 241 (53.2%) were male (Table 1). Hospitals 1, 2, and 3 contributed 16 902 patients (42.4%), 4132 patients (10.4%), and 4528 patients (11.4%) to the entire cohort, respectively. Patient comorbidities varied across the hospitals: patients with hematological malignant neoplasms and transplant recipients were predominantly admitted to hospital 1, and patients with chronic obstructive pulmonary disease and end-stage kidney disease receiving hemodialysis were predominantly admitted to hospital 3.

**Table 1.** Cohort Description for the Entire Cohort of Adult Patients With Sepsis Admitted Between January 2016 and October 2021 at Any of the 10 Hospitals

Feature	Patients, No. (%)				*P* value
	Entire cohort^a^	Hospital 1	Hospital 2	Hospital 3	
Patients	39 893 (100)	16 902 (42.4)	4132 (10.4)	4528 (11.4)	NA
Demographic characteristics					
Age, median (IQR), y	65 (54-74)	61 (50-70)	70 (59-79)	65 (55-76)	<.001
Sex					
Male	21 241 (53.2)	9318 (55.1)	2059 (49.8)	2276 (50.3)	<.001
Female	18 652 (46.8)	7584 (44.9)	2073 (50.2)	2252 (49.7)	
Race and ethnicty^b^					
Black	9112 (22.8)	5024 (29.7)	523 (12.7)	2993 (66.1)	<.001
White	23 982 (60.1)	11 085 (65.6)	3473 (84.1)	1424 (31.4)	<.001
Other	6799 (17.0)	794 (4.7)	132 (3.2)	113 (2.5)	<.001
Comorbidities					
ESKD	4282 (10.7)	1689 (10.0)	500 (12.1)	849 (18.8)	<.001
COPD	8893 (22.3)	3277 (19.4)	869 (21.0)	1389 (30.7)	<.001
Alcoholic cirrhosis	675 (1.7)	372 (2.2)	53 (1.3)	76 (1.7)	<.001
Hematological malignant neoplasm	2635 (6.6)	1969 (11.6)	198 (4.8)	133 (2.9)	<.001
Solid organ transplantation	1630 (4.1)	1381 (8.2)	90 (2.2)	53 (1.2)	<.001
Bacterial pneumonia	5201 (13.0)	2074 (12.3)	685 (16.6)	735 (16.2)	<.001
Other sepsis	18 855 (47.3)	8949 (52.9)	2300 (55.7)	2417 (53.4)	.01
GNB infection	7822 (19.6)	3708 (21.9)	977 (23.6)	1186 (26.2)	<.001
Resistance to antimicrobial drugs	2379 (6.0)	1294 (7.7)	294 (7.1)	287 (6.3)	.01
Severity of illness					
Septic shock	4600 (11.5)	2424 (14.3)	378 (9.1)	282 (6.2)	<.001
Intubation	11 615 (29.1)	4808 (28.4)	885 (21.4)	1752 (38.7)	<.001
Culture specimen					
Blood	1602 (13.7)	623 (13.3)	177 (15.2)	174 (11.7)	.20
Urine	4575 (39.0)	1535 (32.7)	548 (47.2)	551 (36.9)	<.001
Respiratory	2957 (25.2)	1339 (28.5)	185 (15.9)	296 (19.8)	<.001
Other	2598 (22.1)	1201 (25.6)	251 (21.6)	472 (31.6)	<.001
Resistance patterns					
SS	35 343 (88.6)	14 819 (87.7)	3766 (91.1)	3987 (88.1)	<.001
RS	2851 (7.1)	1275 (7.5)	236 (5.7)	303 (6.7)	<.001
RR	1699 (4.3)	808 (4.8)	130 (3.1)	238 (5.3)	<.001
No. of instances/patient, median (IQR)	1 (1-2)	2 (1-3)	1 (1-2)	1 (1-2)	.79

Abbreviations: COPD, chronic obstructive pulmonary disease; ESKD, end-stage kidney disease; GNB, gram-negative bacilli; NA, not applicable; RR, gram-negative bacilli isolates resistant to both ceftriaxone and cefepime; RS, gram-negative bacilli isolates resistant to ceftriaxone and susceptible to cefepime; SS, gram-negative bacilli isolates susceptible to ceftriaxone or culture negative for any gram-negative bacilli.

^a^ The study included all adult patients admitted to 10 hospitals in the Midwest. Only 3 hospitals are illustrated as examples of different patient case mixes.

^b^ Patients verbally reported their race and ethnicity. Any groups reported besides Black and White were classified as other.

We included 49 726 admissions and 85 238 sepsis episodes in the final dataset. Community-onset accounted for 50.7% of the sepsis episodes (43 207 episodes) (Table 2). Hospital-onset sepsis (42 031 episodes [48.3%]) occurred at a median (IQR) of 10 (5-19) days after admission. RS and RR isolates were more common in hospital-onset sepsis compared with community-acquired sepsis (RS: 2389 episodes [5.7%] vs 1667 episodes [3.9%]; RR: 1626 [3.9%] vs 796 [1.8%]). History of previous infections (eg, pneumonia and sepsis) along with history of resistant isolates were more common in the RS and RR groups in community-onset sepsis. RR isolates were cultured from urinary specimens in community-onset sepsis, and from respiratory specimens in hospital-onset sepsis. Previous infections and exposure to antibiotics were associated with the risk of resistant GNB. For example, in community-onset sepsis, 375 RR episodes (47.1%), 420 RS episodes (25.2%) and 3483 of 40 744 (8.5%) SS episodes were among patients with resistance to antimicrobial drugs (P < .001).

**Table 2.** Feature Distribution per Outcome Groups in Community-Onset and Hospital-Onset Sepsis for 85 238 Sepsis Episodes Between January 2016 and October 2021 Across 10 Hospitals

Feature	Patients by isolate, No. (%)				*P* value
	All	SS	RS	RR	
**Community-onset sepsis** ^a^					
No. of sepsis episodes	43 207 (100)	40 744 (94.3)	1667 (3.9)	796 (1.8)	NA
Demographic characteristics					
Age, median (IQR), y	64 (53-74)	64 (53-74)	65 (55-74)	65 (54-75)	<.001
Sex					
Male	22 530 (52.1)	21 212 (52.1)	893 (53.6)	425 (53.4)	.37
Female	20 677 (47.9)	19 532 (47.9)	774 (46.4)	371 (46.6)	
Comorbidities					
ESKD	6295 (14.6)	5862 (14.4)	289 (17.3)	144 (18.1)	<.001
COPD	10 999 (25.5)	10 282 (25.2)	469 (28.1)	248 (31.2)	<.001
Alcoholic cirrhosis	909 (2.1)	864 (2.1)	29 (1.7)	16 (2.0)	.56
Hematological malignant neoplasm	3855 (8.9)	3664 (9.0)	138 (8.3)	53 (6.7)	.05
Solid organ transplantation	2736 (6.3)	2552 (6.3)	127 (7.6)	57 (7.2)	.05
Bacterial pneumonia	7725 (17.9)	6973 (17.1)	500 (30.0)	252 (31.7)	<.001
Other sepsis	26 046 (60.3)	24 053 (59.0)	1329 (79.7)	664 (83.4)	<.001
GNB infections	11 863 (27.5)	10 345 (25.4)	983 (59.0)	535 (67.2)	<.001
Resistance to antimicrobial drugs	4278 (9.9)	3483 (8.5)	420 (25.2)	375 (47.1)	<.001
No history of sepsis, pneumonia, GNB infections, or resistance to antimicrobial drugs	12 842 (29.7)	12 707 (31.2)	96 (5.8)	39 (4.9)	<.001
Severity of illness					
Septic shock	4331 (10)	4142 (10.2)	125 (7.5)	64 (8.0)	<.001
Intubation	11 608 (26.9)	10 999 (27.0)	432 (25.9)	177 (22.2)	.008
Culture specimen					
Blood	1492 (17.8)	1005 (17.0)	317 (19.0)	170 (21.4)	<.001
Urine	3902 (46.7)	3053 (51.7)	555 (33.3)	294 (36.9)	<.001
Respiratory	1006 (12.0)	446 (7.6)	372 (22.3)	188 (23.6)	<.001
Other	1963 (23.5)	1396 (23.7)	423 (25.4)	144 (18.1)	<.001
**Hospital-onset sepsis** ^b^					
No. of sepsis episodes	42 031 (100)	38 016 (9.4)	2389 (5.7)	1626 (3.9)	NA
Demographic characteristics					
Age, median (IQR), y	61 (50-70)	61 (50-70)	63 (54-72)	60 (49-69)	<.001
Sex					
Male	24 172 (57.5)	21 712 (57.1)	1478 (61.9)	982 (60.4)	<.001
Female	17 859 (42.5)	16 304 (42.9)	911 (38.1)	644 (39.6)	
Comorbidities					
ESKD	6192 (14.7)	5454 (14.3)	399 (16.7)	339 (2.8)	<.001
COPD	8763 (2.8)	7829 (2.6)	530 (22.2)	404 (24.8)	<.001
Alcoholic cirrhosis	698 (1.7)	646 (1.7)	27 (1.1)	25 (1.5)	.10
Hematological malignant neoplasm	4611 (11.0)	4381 (11.5)	112 (4.7)	118 (7.3)	<.001
Transplantation	3117 (7.4)	2927 (7.7)	103 (4.3)	87 (5.4)	<.001
Bacterial pneumonia	7989 (19.0)	6579 (17.3)	732 (3.6)	678 (41.7)	<.001
Other sepsis	20 787 (49.5)	18 648 (49.1)	1132 (47.4)	1007 (61.9)	<.001
GNB infections	9431 (22.4)	8032 (21.1)	776 (32.5)	623 (38.3)	<.001
Resistance to antimicrobial drugs	4631 (11.0)	3829 (1.1)	325 (13.6)	477 (29.3)	<.001
No history of pneumonia, sepsis, GNB infections, or resistance to antimicrobial drugs	18321 (43.6)	16863 (44.4)	981 (41.1)	477 (29.3)	<.001
Severity of illness					
Septic shock	5223 (12.4)	4624 (12.2)	349 (14.6)	250 (15.4)	<.001
Intubation	18654 (44.4)	16 550 (43.5)	1256 (52.6)	848 (52.2)	<.001
Culture specimen					
Blood	665 (9.2)	325 (1.2)	222 (9.3)	118 (7.3)	<.001
Urine	1426 (19.8)	876 (27.5)	401 (16.8)	149 (9.2)	<.001
Respiratory	3153 (43.8)	822 (25.8)	1284 (53.7)	1047 (64.4)	<.001
Other	1956 (27.2)	1162 (36.5)	482 (2.2)	312 (19.2)	<.001

Abbreviations: COPD, chronic obstructive pulmonary disease; ESKD, end-stage kidney disease; GNB, gram-negative bacilli; NA, not applicable; RR, gram-negative bacilli isolates resistant to both ceftriaxone and cefepime; RS, gram-negative bacilli isolates resistant to ceftriaxone and susceptible to cefepime; SS, gram-negative bacilli isolates susceptible to ceftriaxone or culture negative for any gram-negative bacilli.

^a^ *Escherichia coli* (36.2% of RR) and *Pseudomonas aeruginosa* (56.6% of RS isolates) were the most common resistant isolates in community-onset sepsis.

^b^ *P aeruginosa* (58.3%) was the most common resistant isolate in hospital-onset sepsis.

**Figure 1.**
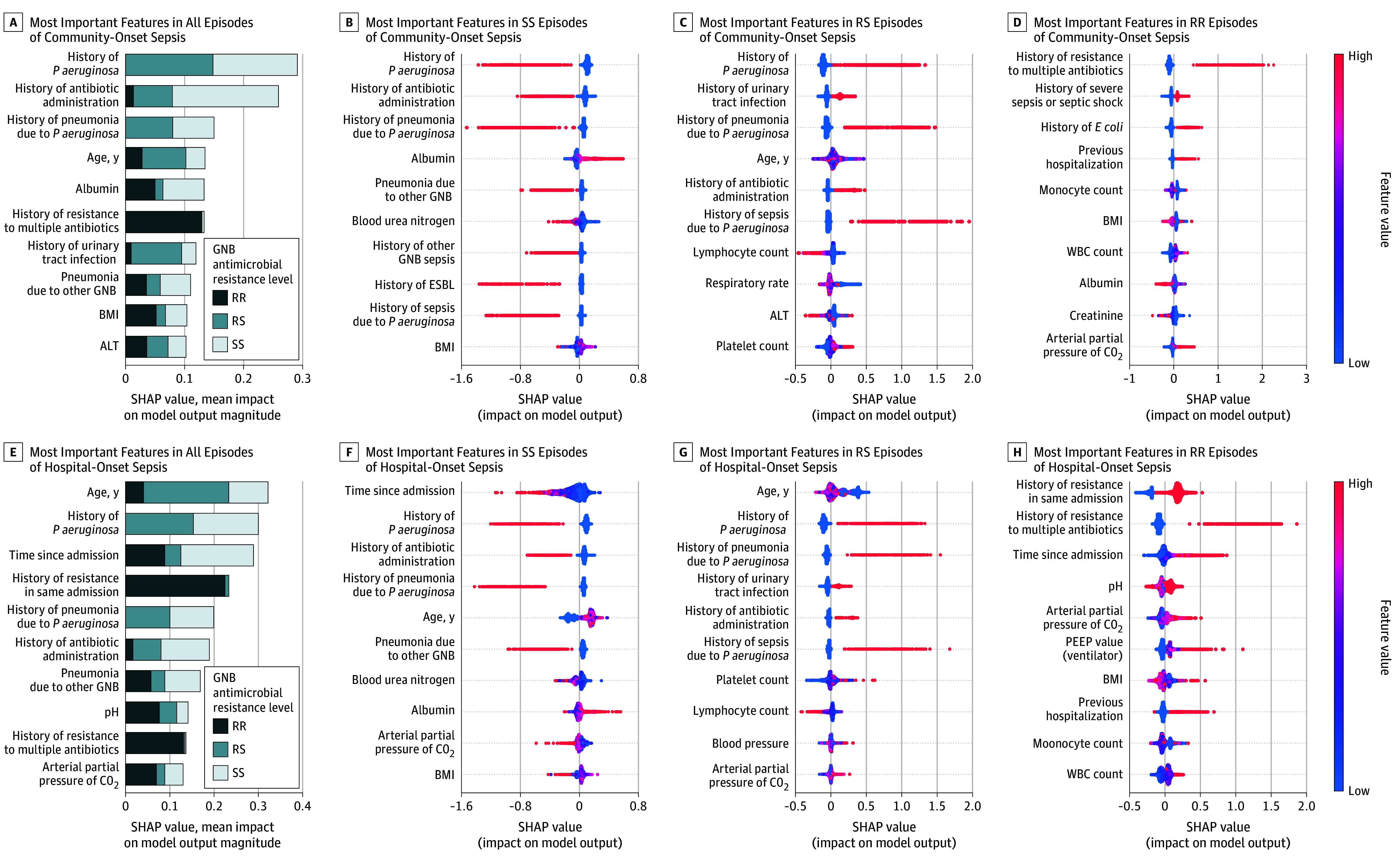
Ten Most Important Features for Gradient-Boosting Model Performance in Community-Onset and Hospital-Onset Sepsis Feature importance in descending order is shown on the vertical axis. Plotted points represent the distinct value for each infection analyzed with color coding if the value was high (red) or low (blue). Shapley additive explanation (SHAP) values displayed on the horizontal axis reflect whether a value was associated with increased or decreased risk. ALT indicates alanine transaminase; BMI, body mass index; *E coli*, *Escherichia coli*; ESBL, extended spectrum beta-lactamase; GNB, gram-negative bacilli; *P aeruginosa*, *Pseudomonas aeruginosa*; PEEP, positive end-expiratory pressure; RR, ceftriaxone-resistant and cefepime-resistant gram-negative bacilli; RS, ceftriaxone-resistant and cefepime-susceptible gram-negative bacilli; SS, ceftriaxone- and cefepime-susceptible gram-negative bacilli; and WBC, white blood cell.

The deep learning risk stratification model for RS and RR episodes in community-onset sepsis had AUROCs of 0.84 (95% CI, 0.83-0.84) and 0.81 (95% CI, 0.79-0.83), respectively, and AUPRCs of 0.27 (95% CI, 0.24-0.30) and 0.13 (95% CI, 0.11-0.15), respectively (eFigure 1 in Supplement 1). In hospital-onset sepsis, the AUPRC for RR isolates was 0.16 (95% CI, 0.15-0.17), and the AUPRC for RS isolates was 0.25 (95% CI, 0.23-0.27). In community-onset sepsis, history of *P aeruginosa* infections and history of antibiotic administration were the strongest factors for discriminating RS and RR from SS (Figure 1). History of antibiotic-resistant organisms (ARO) and previous episodes of severe sepsis or septic shock were associated with RR. In hospital-onset sepsis, age and history of *P aeruginosa* infections were the most impactful factors. Time since admission was associated with lower risk of SS and higher risk of RR. Comorbidities and medical history had the highest contribution to model performance in the deep learning and gradient-boosting models (deep learning: AUROC, 0.81 [95% CI, 0.81-0.81]; AUPRC, 0.30 [95% CI, 0.29-0.31]; gradient boosting: AUROC, 0.76 [95% CI, 0.76-0.76]; AUPRC, 0.23 [95% CI, 0.23-0.23]) (data not shown). While indicative of severity, vasopressor use and longitudinal data, such as vital signs and laboratory results, added minimal contributions to GNB resistance prediction.

**Figure 2.**
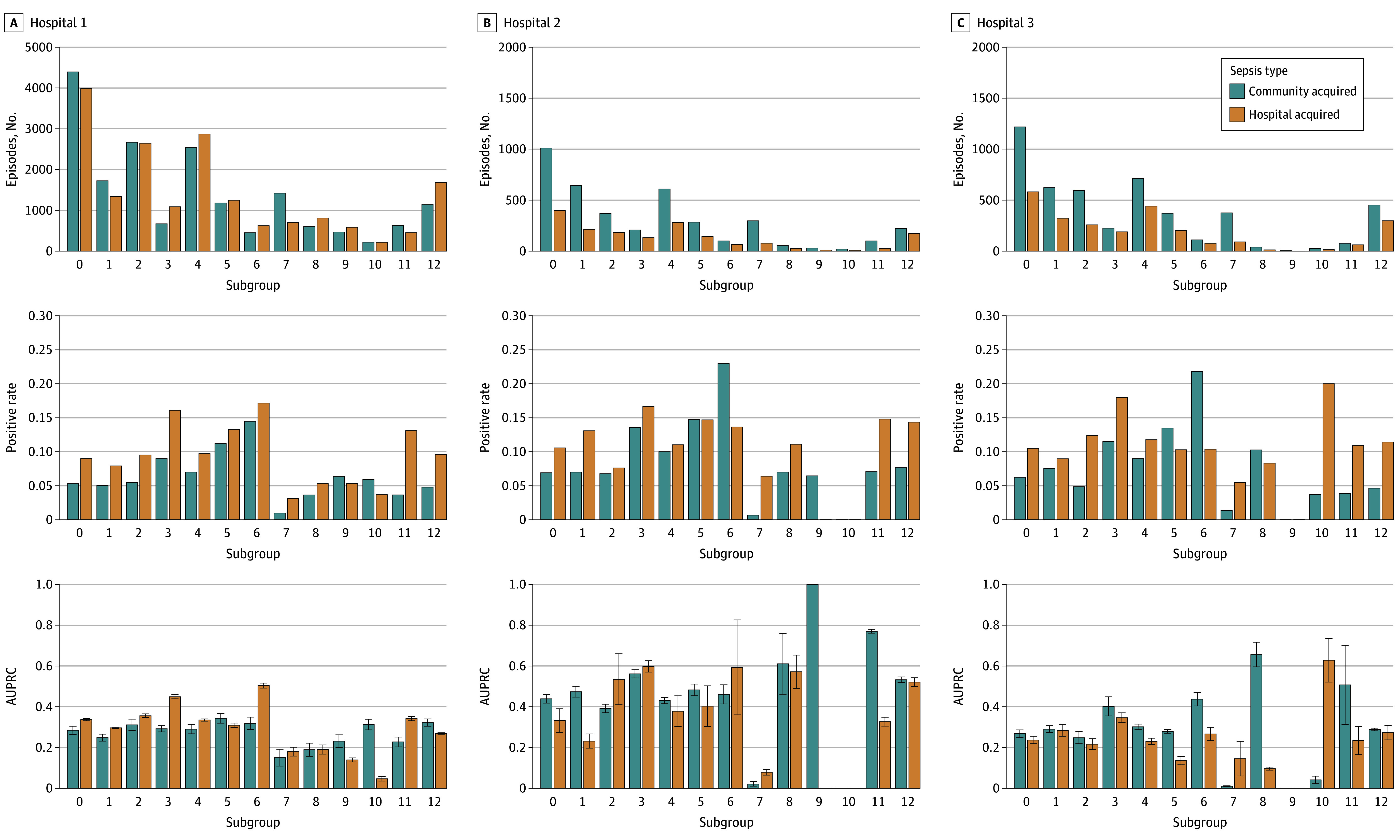
Deep Learning Model Performance Across Patient Subgroups of Interest and Hospitals The number of sepsis episodes and the positive rates of gram-negative bacilli (GNB) isolates resistant to ceftriaxone and susceptible to cefepime as well as GNB isolates resistant to both ceftriaxone and cefepime are shown. C, Whiskers indicate ±1 SD. Subgroups are as follows: 0, entire cohort; 1, patients aged 65 years or older; 2, patients younger than 65 years; 3, patients with history of bacterial pneumonia; 4, patients with history of other sepsis; 5, patients with history of disease-causing GNB such as *Escherichia coli*, *Klebsiella* spp, and *Pseudomonas aeruginosa*; 6, patients with history of antibiotic-resistant microbes; 7, patients with no history of bacterial pneumonia, other sepsis, disease-causing GNB, or antibiotic resistant microbes; 8, patients with hematological malignant neoplasms; 9, patients who underwent transplant; 10, patients with alcoholic cirrhosis; 11, patients with septic shock; and 12, patients who underwent intubation. AUPRC indicates area under the precision recall curve.

**Figure 3.**
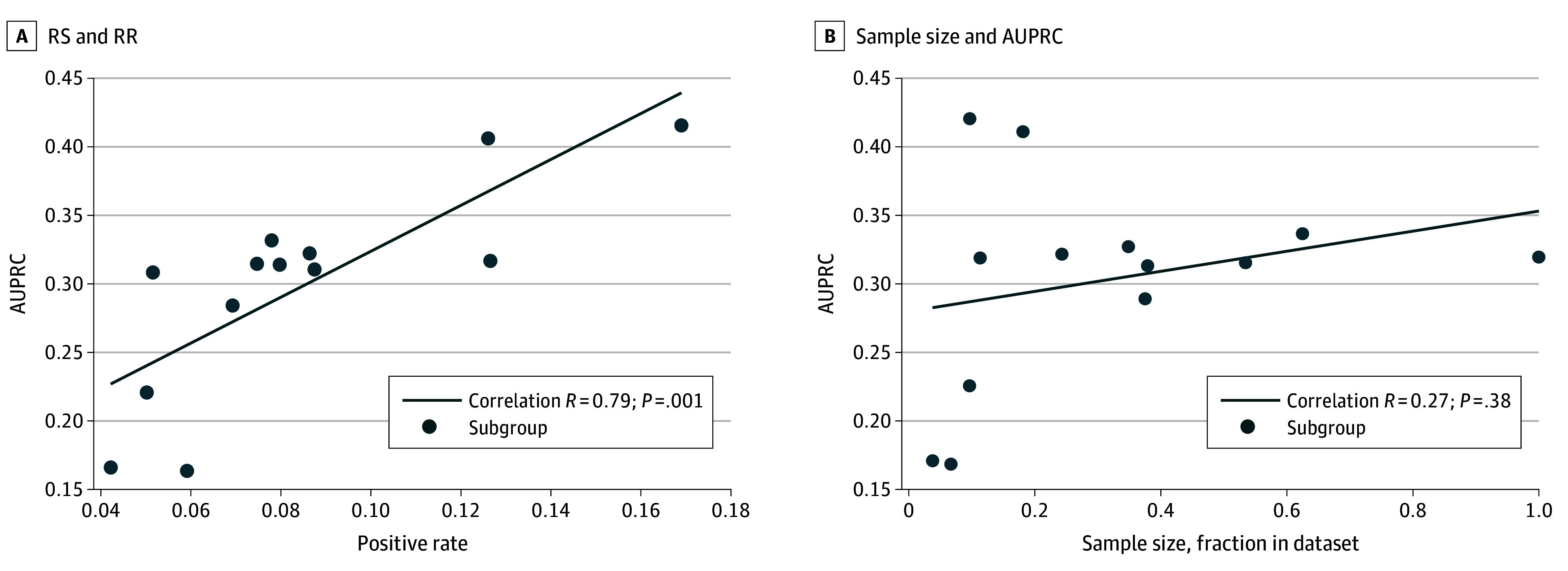
Correlation Plots Between Prevalence Rate of Gram-Negative Bacilli (GNB) Isolates Resistant to Ceftriaxone and Susceptible to Cefepime (RS) and GNB Isolates Resistant to Both Ceftriaxone and Cefepime (RR) and Sample Size With Area Under the Precision Recall Curve (AUPRC) Across Patient Subgroups Analyses were repeated after excluding patient subgroups that contributed less than 0.2 fraction in the dataset without improved *R* coefficient or *P* value.

The deep learning model performance varied across hospitals (Figure 2). Patient subgroups contributed different fractions to the hospital case mixes, and these subgroups had varied prevalence rates of RS and RR GNB and AUPRCs. For example, patients with hematological malignant neoplasms had RS and RR prevalence rates from 3.6% (hospital 1) to 10.3% (hospital 3) in community-onset sepsis and from 5.3% (hospital 1) to 11.1% (hospital 2) in hospital-onset sepsis, with wide variation in AUPRC. When analyzing the subgroups across the entire cohort, projections of RS and RR microbial etiologies in community-onset sepsis had the highest AUPRC of 0.36 (95% CI, 0.35-0.37) in patients with history of ARO, followed by patients with history of bacterial pneumonia or other infections caused by GNB (eFigure 2 in Supplement 1). Projections of RS and RR microbial etiologies in hospital-onset sepsis had the highest AUPRCs of 0.47 (95% CI, 0.43-0.51) and 0.45 (95% CI, 0.44-0.46), respectively, in patients with history of ARO and history of bacterial pneumonia. The deep learning model AUPRC across patient subgroups of interest correlated with the RS and RR prevalence rates (*R* = 0.79; *P* < .001) (Figure 3). The subgroup sample sizes did not correlate with AUPRC.

## Discussion

This study analyzed a large and diverse dataset of patients across urban and rural areas of the greater St Louis region in Missouri and Illinois. The data show that the risk for resistant GNB in sepsis differs significantly between hospital- and community-onset sepsis and across hospitals. The risk appears to depend on the RS and RR prevalence rates in hospitals and subgroups of patients and does not solely depend on patient comorbidities. The most relevant features associated with ceftriaxone- and cefepime-resistant GNB were history of several infections and previous exposure to antibiotics. Previous risk stratification tools only included patients with positive cultures and were developed at large academic centers with high prevalence rates of resistance. The prevalence rates in published derivation cohorts (20%-40%) varied widely from broad observational studies.^2,5,19,20,21,22^ The unintended consequence was widespread overuse of broad-spectrum antibiotics, which may have detrimental effects at the population and patient levels.^2^ Our study tested the translation of a generalized model and included patients with both positive and negative culture results. Our approach tries to correct limitations in previous studies and to reassess the need for uniform broad-spectrum antibiotics in sepsis based on risk factors.^23^ We focused on AUPRC, which evaluates the average positive predictive value over all sensitivity thresholds. In the setting of a highly imbalanced dataset with very few infections caused by resistant GNB, AUPRC measures the model’s ability to project the positive cases.

Risk stratification models are critical in the treatment of sepsis. Risk factors extracted from observational data contribute to the background of sepsis treatment guidelines. Our analysis supported accepted risk factors^24,25,26,27^ and showed that patients with previous sepsis, pneumonia, other infections caused by GNB, and known ARO had higher prevalence rates of resistant GNB, but these rates varied across the hospitals. Current sepsis guidelines suggest using 2 antimicrobials for empirical treatment in patients with septic shock and at high risk for antibiotic-resistant GNB. High-risk factors include previous infections with resistant GNB, recent use of antibiotics, recent hospitalization, and local prevalence resistance rates.^1^ Clinicians follow sepsis guidelines although the risk stratification models were not developed in house, which can lead to erroneous use of antibiotics. A national study in the Veterans Affairs population reported that only 0.9% of patient samples retrieved ARO GNB, but 76% of these patients were flagged as candidates for double GNB coverage in pneumonia.^28^

Patient case mix or patient-level factors have been suggested as necessary risk-adjustment variables for antimicrobial use monitoring.^29^ The standardized antimicrobial administration ratio metric was developed by the CDC to track antimicrobial use. It is reported as the observed-to-predicted ratio and is adjusted largely per hospital and unit type.^30^ Previous studies indicated a need for patient-level adjustments.^31,32^ The assumption is that certain patient subgroups experience infections caused by resistant GNB, and broader empirical therapy is accepted. Implicitly the assumption is that these subgroups, such as patients with hematological malignant neoplasms, carry the same risk across all hospitals. Our results suggest that patient-level comorbidities may not fully explain resistant GNB infections and suggest that local prevalence of resistance rates impact risk stratification models that guide clinicians’ empirical choices. Patients with hematological malignant neoplasms, transplantation, or septic shock will fall into different risk categories depending on the institution where they receive care. The performance metrics of risk stratification models varied greatly across hospitals, even for patient subgroups without previous infections or known antibiotic exposure. Similar to antibiograms, rates of antimicrobial resistance proximal to the sepsis episode being treated would be most informative.

Our study extends the knowledge gained from generalized early warning systems for sepsis. The Epic Sepsis Model, derived on data from 405 000 patient encounters across 3 health care systems, was integrated into the clinical EHR system to alert clinicians when patients develop sepsis.^33^ Hundreds of hospitals adopted the system into clinical workflow before it failed external validation.^34^ The model labeled 18% of all patients admitted to University of Michigan Health as developing sepsis but failed to detect sepsis in 67% of patients with sepsis. Our study encourages institutions to develop their own risk stratification models to identify patients at risk for resistant GNB similar to maintaining local antibiograms.

### Limitations

Our study had limitations, including those inherent to retrospective analyses based on EHR data. We did not include potential confounders, such as nursing home residence, outpatient prescriptions, or duration of central lines and devices. Patients admitted from nursing homes were classified as community-onset infections if the infections were present at admission. A large study that captures difficult-to-extract variables, such as nursing home residence, which are not usually stored in a structured data format, is nearly impossible. We relied on sophisticated deep learning algorithms to harness and learn from the collective EHR data with the least assumptions. We mapped 16 926 comorbidities, more than 9 million data points for vital signs and 8 million laboratory results. We defined broad patient subgroups of interest to facilitate a broader understanding. Our goal was to investigate the variation in the performance of a static model across hospitals and patient subgroups that reflect differences in patient case mix. To avoid model brittleness, defined as the failure to generalize to new data, we developed a general model and tested it on holdout data from the same hospitals and period. The robustness of the model against subgroup distribution heterogeneity may be improved using domain generalization approaches or group-fairness classification approaches, which we are considering for future work.^35,36^

## Conclusions

In this cohort study of patients with sepsis admitted to 10 Midwest hospitals, the performance of the risk stratification model was associated with the prevalence rates of resistant GNB and varied across hospitals and patient subgroups. These results do not support widespread use of generalized models. Future efforts to benchmark antimicrobial use may need to include community and hospital resistance rates (warranting availability of microbiology data) to accompany patient-level variables.

## Notes

Supplement 1. **eTable.** Data Description and Preprocessing for Adult Sepsis Cohort 2016 to 2021 in 10 Hospitals **eFigure 1.** Testing AUROC and AUPRC of Deep Learning Model in Community-Onset and Hospital-Onset Sepsis **eFigure 2.** Deep Learning Model Performance Across Patient Subgroups of Interest for the Entire Cohort

Supplement 2.
Data Sharing Statement

## References

[1] Evans L, Rhodes A, Alhazzani W, . Surviving Sepsis Campaign: international guidelines for management of sepsis and septic shock 2021. Crit Care Med. 2021;49(11):e1063-e1143. doi:10.1097/CCM.0000000000005337 34605781

[2] Rhee C, Kadri SS, Dekker JP, ; CDC Prevention Epicenters Program. Prevalence of antibiotic-resistant pathogens in culture-proven sepsis and outcomes associated with inadequate and broad-spectrum empiric antibiotic use. JAMA Netw Open. 2020;3(4):e202899. doi:10.1001/jamanetworkopen.2020.2899 32297949 PMC7163409

[3] Seymour CW, Gesten F, Prescott HC, . Time to treatment and mortality during mandated emergency care for sepsis. N Engl J Med. 2017;376(23):2235-2244. doi:10.1056/NEJMoa1703058 28528569 PMC5538258

[4] Kadri SS, Lai YL, Warner S, ; forming the National Insititutes of Health Antimicrobial Resistance Outcomes Research Initiative (NIH-ARORI). Inappropriate empirical antibiotic therapy for bloodstream infections based on discordant in-vitro susceptibilities: a retrospective cohort analysis of prevalence, predictors, and mortality risk in US hospitals. Lancet Infect Dis. 2021;21(2):241-251. doi:10.1016/S1473-3099(20)30477-1 32916100 PMC7855478

[5] Ohnuma T, Chihara S, Costin B, . Association of appropriate empirical antimicrobial therapy with in-hospital mortality in patients with bloodstream infections in the US. JAMA Netw Open. 2023;6(1):e2249353. doi:10.1001/jamanetworkopen.2022.49353 36598788 PMC9857618

[6] MacFadden DR, Coburn B, Shah N, . Decision-support models for empiric antibiotic selection in Gram-negative bloodstream infections. Clin Microbiol Infect. 2019;25(1):108.e1-108.e7. doi:10.1016/j.cmi.2018.03.029 29705558

[7] von Elm E, Altman DG, Egger M, Pocock SJ, Gøtzsche PC, Vandenbroucke JP; STROBE Initiative. The Strengthening the Reporting of Observational Studies in Epidemiology (STROBE) statement: guidelines for reporting observational studies. Ann Intern Med. 2007;147(8):573-577. doi:10.7326/0003-4819-147-8-200710160-00010 17938396

[8] Stevens LM, Mortazavi BJ, Deo RC, Curtis L, Kao DP. Recommendations for reporting machine learning analyses in clinical research. Circ Cardiovasc Qual Outcomes. 2020;13(10):e006556. doi:10.1161/CIRCOUTCOMES.120.006556 33079589 PMC8320533

[9] Pineau J, Vincent-Lamarre P, Sinha K, . Improving reproducibility in machine learning research (a report from the NeurIPS 2019 Reproducibility Program). arXiv. Preprint posted online December 30, 2020. doi:10.48550/arXiv.2003.12206

[10] Rhee C, Zhang Z, Kadri SS, ; CDC Prevention Epicenters Program. Sepsis surveillance using Adult Sepsis Events Simplified eSOFA criteria versus Sepsis-3 Sequential Organ Failure Assessment criteria. Crit Care Med. 2019;47(3):307-314. doi:10.1097/CCM.0000000000003521 30768498 PMC6383796

[11] Clinical and Laboratory Standards Institute. Performance standards for antimicrobial susceptibility testing, 34th Edition. Accessed May 23, 2024. https://clsi.org/standards/products/microbiology/documents/m100/

[12] National Bureau of Economic Research. *ICD-9-CM* to and from *ICD-10-CM* and *ICD-10-PCS* crosswalk or general equivalence mappings. Accessed May 23, 2024. https://www.nber.org/research/data/icd-9-cm-and-icd-10-cm-and-icd-10-pcs-crosswalk-or-general-equivalence-mappings

[13] Štrumbelj E, Kononenko I. Explaining prediction models and individual predictions with feature contributions. Knowl Inf Syst. 2014;41(3):647-665. doi:10.1007/s10115-013-0679-x

[14] Chen T, Guestrin C. XGBoost: A scalable tree boosting system. In: Proceedings of the 22nd ACM SIGKDD International Conference on Knowledge Discovery and Data Mining. KDD ’16. Association for Computing Machinery; 2016:785-794. doi:10.1145/2939672.2939785

[15] Vaswani A, Shazeer N, Parmar N, . Attention is all you need. arXiv. Preprint posted online August 1, 2023. doi:10.48550/arXiv.1706.03762

[16] Grover A, Leskovec J. node2vec: Scalable feature learning for networks. In: Proceedings of the 22nd ACM SIGKDD International Conference on Knowledge Discovery and Data Mining. KDD ’16. Association for Computing Machinery; 2016:855-864. doi:10.1145/2939672.2939754 PMC5108654 27853626

[17] Chawla NV, Bowyer KW, Hall LO, Kegelmeyer WP. SMOTE: synthetic minority over-sampling technique. J Artif Intell Res. 2002;16:321-357. doi:10.1613/jair.953

[18] Lin TY, Goyal P, Girshick R, He K, Dollár P. Focal loss for dense object detection. arXiv. Preprint posted online February 7, 2018. doi:10.48550/arXiv.1708.02002 30040631

[19] Lodise TP, Bonine NG, Ye JM, Folse HJ, Gillard P. Development of a bedside tool to predict the probability of drug-resistant pathogens among hospitalized adult patients with gram-negative infections. BMC Infect Dis. 2019;19(1):718. doi:10.1186/s12879-019-4363-y 31412809 PMC6694572

[20] Vasudevan A, Mukhopadhyay A, Li J, Yuen EGY, Tambyah PA. A prediction tool for nosocomial multi-drug resistant Gram-negative Bacilli infections in critically ill patients—prospective observational study. BMC Infect Dis. 2014;14:615. doi:10.1186/s12879-014-0615-z 25420613 PMC4252002

[21] Shorr AF, Zilberberg MD, Micek ST, Kollef MH. Prediction of infection due to antibiotic-resistant bacteria by select risk factors for health care-associated pneumonia. Arch Intern Med. 2008;168(20):2205-2210. doi:10.1001/archinte.168.20.2205 19001196

[22] Shorr AF, Zilberberg MD, Reichley R, . Validation of a clinical score for assessing the risk of resistant pathogens in patients with pneumonia presenting to the emergency department. Clin Infect Dis. 2012;54(2):193-198. doi:10.1093/cid/cir813 22109951

[23] Pak TR, Young J, McKenna CS, . Risk of misleading conclusions in observational studies of time-to-antibiotics and mortality in suspected sepsis. Clin Infect Dis. 2023;77(11):1534-1543. doi:10.1093/cid/ciad450 37531612 PMC10686960

[24] Vazquez-Guillamet MC, Vazquez R, Micek ST, Kollef MH. Predicting resistance to piperacillin-tazobactam, cefepime and meropenem in septic patients with bloodstream infection due to Gram-negative bacteria. Clin Infect Dis. 2017;65(10):1607-1614. doi:10.1093/cid/cix612 29020294

[25] Goodman KE, Lessler J, Cosgrove SE, ; Antibacterial Resistance Leadership Group. A clinical decision tree to predict whether a bacteremic patient is infected with an extended-spectrum β-lactamase-producing organism. Clin Infect Dis. 2016;63(7):896-903. doi:10.1093/cid/ciw425 27358356 PMC5019284

[26] Stone TJ, DeWitt M, Johnson JW, . Analysis of infections among patients with historical culture positive for extended-spectrum beta-lactamase (ESBL)-producing *Escherichia coli* or *Klebsiella pneumoniae*: is ESBL-targeted therapy always needed? Antimicrob Steward Healthc Epidemiol. 2023;3(1):e47. doi:10.1017/ash.2022.363 36970424 PMC10031583

[27] Denkel LA, Maechler F, Schwab F, . Infections caused by extended-spectrum β-lactamase-producing *Enterobacterales* after rectal colonization with ESBL-producing *Escherichia coli* or *Klebsiella pneumoniae*. Clin Microbiol Infect. 2020;26(8):1046-1051. doi:10.1016/j.cmi.2019.11.025 31809805

[28] Bostwick AD, Jones BE, Paine R, Goetz MB, Samore M, Jones M. Potential impact of hospital-acquired pneumonia guidelines on empiric antibiotics: an evaluation of 113 Veterans Affairs medical centers. Ann Am Thorac Soc. 2019;16(11):1392-1398. doi:10.1513/AnnalsATS.201902-162OC 31385720 PMC6945471

[29] Shively NR, Morgan DJ. The CDC antimicrobial use measure is not ready for public reporting or value-based programs. Antimicrob Steward Healthc Epidemiol. 2023;3(1):e77. doi:10.1017/ash.2023.143 37113208 PMC10127230

[30] van Santen KL, Edwards JR, Webb AK, . The Standardized Antimicrobial Administration Ratio: a new metric for measuring and comparing antibiotic use. Clin Infect Dis. 2018;67(2):179-185. doi:10.1093/cid/ciy075 29409000

[31] Goodman KE, Pineles L, Magder LS, . Electronically available patient claims data improve models for comparing antibiotic use across hospitals: results from 576 US facilities. Clin Infect Dis. 2021;73(11):e4484-e4492. doi:10.1093/cid/ciaa1127 32756970 PMC8662758

[32] Yu KC, Moisan E, Tartof SY, . Benchmarking inpatient antimicrobial use: a comparison of risk-adjusted observed-to-expected ratios. Clin Infect Dis. 2018;67(11):1677-1685. doi:10.1093/cid/ciy354 29688279

[33] Bennett T, Russell S, King J, . Accuracy of the Epic sepsis prediction model in a regional health system. arXiv. Preprint posted online February 19, 2019. doi:10.48550/arXiv.1902.07276

[34] Wong A, Otles E, Donnelly JP, . External validation of a widely implemented proprietary sepsis prediction model in hospitalized patients. JAMA Intern Med. 2021;181(8):1065-1070. doi:10.1001/jamainternmed.2021.2626 34152373 PMC8218233

[35] Wu Z, Yao H, Liebovitz DM, Sun J. An iterative self-learning framework for medical domain generalization. SlidesLive. Accessed September 5, 2024. https://slideslive.com/embed/presentation/39009312?js_embed_version=3&embed_init_token=eyJhbGciOiJIUzI1NiJ9.eyJpYXQiOjE3MjU1NzAwNDgsImV4cCI6MTcyNTY5OTY0OCwidSI6eyJ1dWlkIjoiMDU2NjM3MGYtYzg3Mi00ZmQyLTg1MGItNWRkYTBjOThjODI1IiwiaSI6bnVsbCwiZSI6bnVsbCwibSI6ZmFsc2V9LCJkIjoibmlwcy5jYyJ9.NlSb7EBcx-nMVS0GDt2oT3xEnFmg0p1RYXMoeAkNhuc&embed_parent_url=https%3A%2F%2Fnips.cc%2Fvirtual%2F2023%2Fposter%2F71775&embed_origin=https%3A%2F%2Fnips.cc&embed_container_id=presentation-embed-39009312&auto_load=true&auto_play=false&zoom_ratio=&disable_fullscreen=false&locale=en&vertical_enabled=true&vertical_enabled_on_mobile=false&allow_hidden_controls_when_paused=true&fit_to_viewport=true&custom_user_id=&user_uuid=0566370f-c872-4fd2-850b-5dda0c98c825

[36] Kim JS, Chen J, Talwalkar A. FACT: a diagnostic for group fairness trade-offs. arXiv. Preprint posted online April 7, 2020. doi:10.48550/arXiv.2004.03424

