# Supplementary material for: Performance of Risk Models for Antimicrobial Resistance in Adult Patients With Sepsis
Source: JAMA Netw Open. 2024 Nov 7;7(11):e2443658. doi: 10.1001/jamanetworkopen.2024.43658 (PMC11544496; doi:10.1001/jamanetworkopen.2024.43658)
Supplement: Supplement 1. — eTable. Data Description and Preprocessing for Adult Sepsis Cohort 2016 to 2021 in 10 Hospitals eFigure 1. Testing AUROC and AUPRC of Deep Learning Model in Community-Onset and Hospital-Onset Sepsis eFigure 2. Deep Learning Model Performance Across Patient Subgroups of Interest for the Entire Cohort [file jamanetwopen-e2443658-s001.pdf]

Supplemental Online Content

Vazquez Guillamet MC, Liu H, Atkinson A, Fraser VJ, Lu C, Kollef MH. Performance of risk models for antimicrobial resistance in adult patients with sepsis. *JAMA Netw Open.* 2024;7(11):e2443658. doi:10.1001/jamanetworkopen.2024.43658

eTable. Data Description and Preprocessing for Adult Sepsis Cohort 2016 to 2021 in 10 Hospitals

eFigure 1. Testing AUROC and AUPRC of Deep Learning Model in Community-Onset and Hospital-Onset Sepsis

eFigure 2. Deep Learning Model Performance Across Patient Subgroups of Interest for the Entire Cohort

This supplemental material has been provided by the authors to give readers additional information about their work.

© 2024 Vazquez Guillamet MC et al. *JAMA Network Open.*

eTable. Data Description and Preprocessing for Adult Sepsis Cohort 2016 to 2021 in 10 Hospitals

Data modality	Variable	Data type	Category	Total records	Availability rate	Imputation	Preprocessing	Encoded dimensions
Static	Race	Categorical	4	85615	99.87%	Missing as new category	One-hot encoding	5
	Sex	Categorical	2	85712	99.98%	Missing as new category	One-hot encoding	3
	Hospital	Categorical	10	85728	100%		One-hot encoding	10
	Age	Continuous		85728	100%		Standardization	1
	Time since admission	Continuous		85728	100%		Standardization	1
	Intubation	Binary		85728	100%			1
	History of resistance	Binary		85728	100%			1
	History of hospitalization	Binary		85728	100%			1
	Community-acquired pneumonia	Binary		85728	100%			1
	Hospital-acquired pneumonia	Binary		85728	100%			1
	Vasopressors	List of categorical	8	85728	100%		Multi-hot encoding	8
	Antibiotics	List of categorical	93	85728	100%		Multi-hot encoding	93
Longitudinal (Vital)	Respirations	Continuous		1084802	99.14%	Last observation	Standardization	1 x 30

© 2024 Vazquez Guillamet MC et al. *JAMA Network Open.*

Data modality	Variable	Data type	Category	Total records	Availability rate	Imputation	Preprocessing	Encoded dimensions
	Oxygen saturation, pulse oximetry SpO2	Continuous		1003392	90.18%	Last observation	Standardization	1 x 30
	Pulse	Continuous		747849	73.22%	Last observation	Standardization	1 x 30
	Blood pressure, systolic	Continuous		603855	71.86%	Last observation	Standardization	1 x 30
	Blood pressure, diastolic	Continuous		603856	71.86%	Last observation	Standardization	1 x 30
	Temperature	Continuous		647657	71.53%	Last observation	Standardization	1 x 30
	Body mass index	Continuous		114358	69.94%	Last observation	Standardization	1 x 30
	Blood pressure, mean	Continuous		389570	57.33%	Last observation	Standardization	1 x 30
	Oxygen (O2) therapy, flow	Continuous		272572	45.02%	Last observation	Standardization	1 x 30
	Oxygen (O2), inspired fraction (FiO2)	Continuous		333331	34.12%	Last observation	Standardization	1 x 30
	VentilatorPEEP value	Continuous		208296	24.66%	Last observation	Standardization	1 x 30
	Blood pressure, diastolic, head of bed 30 degrees	Continuous		219952	23.44%	Last observation	Standardization	1 x 30
	Temperature, oral	Continuous		215800	22.41%	Last observation	Standardization	1 x 30
	Pulse, automated	Continuous		251173	20.64%	Last observation	Standardization	1 x 30
	Respirations, vent	Continuous		181767	20.51%	Last observation	Standardization	1 x 30

© 2024 Vazquez Guillamet MC et al. *JAMA Network Open.*

Data modality	Variable	Data type	Category	Total records	Availability rate	Imputation	Preprocessing	Encoded dimensions
	Oxygen saturation, pulse oximetry	Continuous		100034	9.27%	Last observation	Standardization	1 x 30
	Blood pressure, systolic, arterial catheter	Continuous		99704	8.85%	Last observation	Standardization	1 x 30
	Blood pressure, diastolic, arterial catheter	Continuous		99700	8.85%	Last observation	Standardization	1 x 30
	Temperature, bladder	Continuous		71354	6.04%	Last observation	Standardization	1 x 30
	Temperature, temp-2	Continuous		29417	3.25%	Last observation	Standardization	1 x 30
	Temperature, core	Continuous		15997	1.55%	Last observation	Standardization	1 x 30
	Temperature, esophageal	Continuous		10374	1.19%	Last observation	Standardization	1 x 30
Longitudinal (Lab)	WBCS	Continuous		315600	97.77%	Last observation	Standardization	1 x 30
	Hematocrit	Continuous		314286	97.73%	Last observation	Standardization	1 x 30
	Hemoglobin	Continuous		303624	97.56%	Last observation	Standardization	1 x 30
	Platelets	Continuous		298315	97.05%	Last observation	Standardization	1 x 30
	Carbon dioxide	Continuous		300646	95.02%	Last observation	Standardization	1 x 30
	Urea nitrogen (BUN)	Continuous		312093	95.01%	Last observation	Standardization	1 x 30
	Calcium	Continuous		300102	95.00%	Last observation	Standardization	1 x 30
	Sodium	Continuous		313848	94.99%	Last observation	Standardization	1 x 30

© 2024 Vazquez Guillamet MC et al. *JAMA Network Open.*

Data modality	Variable	Data type	Category	Total records	Availability rate	Imputation	Preprocessing	Encoded dimensions
	Chloride	Continuous		307148	94.85%	Last observation	Standardization	1 x 30
	Glucose	Continuous		316438	94.81%	Last observation	Standardization	1 x 30
	Creatinine	Continuous		300104	94.49%	Last observation	Standardization	1 x 30
	Potassium, plasma	Continuous		304037	94.07%	Last observation	Standardization	1 x 30
	Neutrophils, absolute	Continuous		197292	87.83%	Last observation	Standardization	1 x 30
	Lymphocytes, absolute	Continuous		197302	87.83%	Last observation	Standardization	1 x 30
	Monocytes, absolute	Continuous		197240	87.81%	Last observation	Standardization	1 x 30
	Basophils, absolute	Continuous		165937	82.95%	Last observation	Standardization	1 x 30
	Eosinophils, absolute	Continuous		168604	82.81%	Last observation	Standardization	1 x 30
	Albumin	Continuous		152283	82.42%	Last observation	Standardization	1 x 30
	Bilirubin	Continuous		133700	79.81%	Last observation	Standardization	1 x 30
	Alanine transaminase (ALT)	Continuous		131385	79.48%	Last observation	Standardization	1 x 30
	Alkaline phosphatase	Continuous		131369	79.40%	Last observation	Standardization	1 x 30
	PH	Continuous		162934	65.82%	Last observation	Standardization	1 x 30
	Lactic acid	Continuous		96183	58.37%	Last observation	Standardization	1 x 30
	PCO2	Continuous		130914	43.45%	Last observation	Standardization	1 x 30

© 2024 Vazquez Guillamet MC et al. *JAMA Network Open.*

Data modality	Variable	Data type	Category	Total records	Availability rate	Imputation	Preprocessing	Encoded dimensions
	PO2	Continuous		130880	43.38%	Last observation	Standardization	1 x 30
	Potassium	Continuous		66360	24.66%	Last observation	Standardization	1 x 30
	Anion gap	Continuous		24234	10.11%	Last observation	Standardization	1 x 30
	Aspartate transaminase (AST)	Continuous		11836	8.59%	Last observation	Standardization	1 x 30
	Bicarbonate	Continuous		8625	4.40%	Last observation	Standardization	1 x 30
	Magnesium	Continuous		9624	3.90%	Last observation	Standardization	1 x 30
	Troponin I	Continuous		2344	2.00%	Last observation	Standardization	1 x 30
	NT-pro B-type natriuretic peptide (BNP)	Continuous		1761	1.88%	Last observation	Standardization	1 x 30
	B-type natriuretic peptide (BNP)	Continuous		1588	1.87%	Last observation	Standardization	1 x 30
	Calcium, ionized	Continuous		2770	1.36%	Last observation	Standardization	1 x 30
	Amylase	Continuous		836	0.95%	Last observation	Standardization	1 x 30
	Lactate dehydrogenase	Continuous		769	0.66%	Last observation	Standardization	1 x 30
	Cortisol	Continuous		521	0.58%	Last observation	Standardization	1 x 30
	Reticulocyte count, absolute	Continuous		337	0.33%	Last observation	Standardization	1 x 30
	PH, venous	Continuous		283	0.30%	Last observation	Standardization	1 x 30

© 2024 Vazquez Guillamet MC et al. *JAMA Network Open.*

Data modality	Variable	Data type	Category	Total records	Availability rate	Imputation	Preprocessing	Encoded dimensions
	PO2, venous	Continuous		252	0.27%	Last observation	Standardization	1 x 30
	Oxyhemoglobin, venous	Continuous		453	0.24%	Last observation	Standardization	1 x 30
	Schistocytes	Continuous		108	0.10%	Last observation	Standardization	1 x 30

© 2024 Vazquez Guillamet MC et al. *JAMA Network Open.*

eFigure 1. Testing AUROC and AUPRC of Deep Learning Model in Community-Onset (A, B) and Hospital-Onset (C, D) Sepsis

A, B. Community-onset sepsis

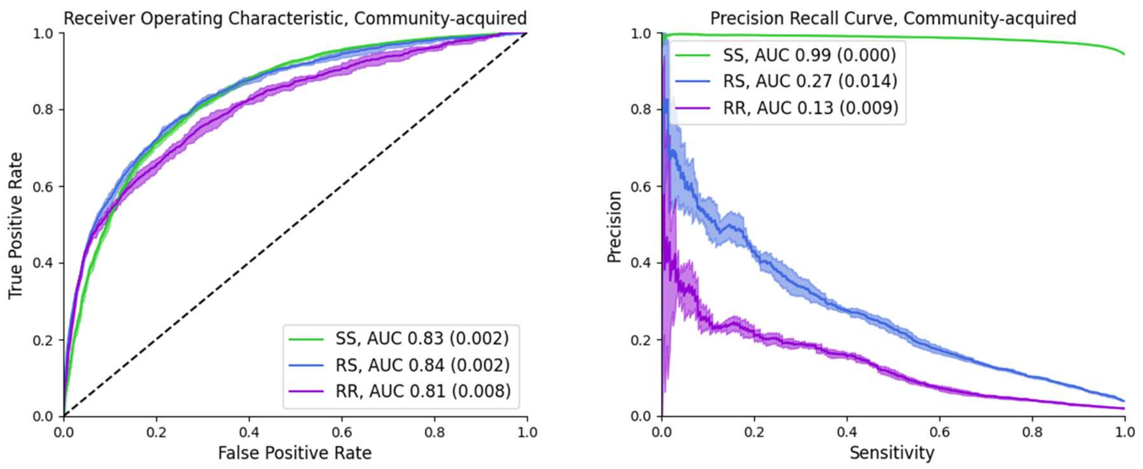

C, D. Hospital-onset sepsis

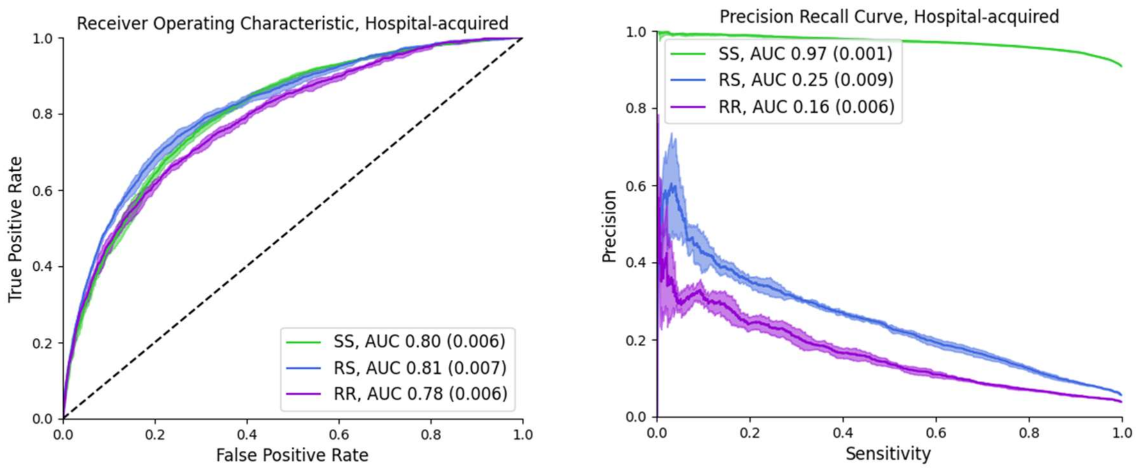

Highlighted area is ±1 standard deviation around the mean performance.
Abbreviations. AUC: Area under curve; AUROC: area under receiver operating characteristic; AUPRC: area under the precision recall curve; SS: Ceftriaxone- and cefepime-susceptible Gram-negative bacilli; RS: Ceftriaxone-resistant and cefepime-susceptible Gram-negative bacilli; RR: Ceftriaxone-resistant and cefepime-resistant Gram-negative bacilli.

© 2024 Vazquez Guillamet MC et al. *JAMA Network Open.*

eFigure 2. Deep Learning Model Performance Across Patient Subgroups of Interest for the Entire Cohort

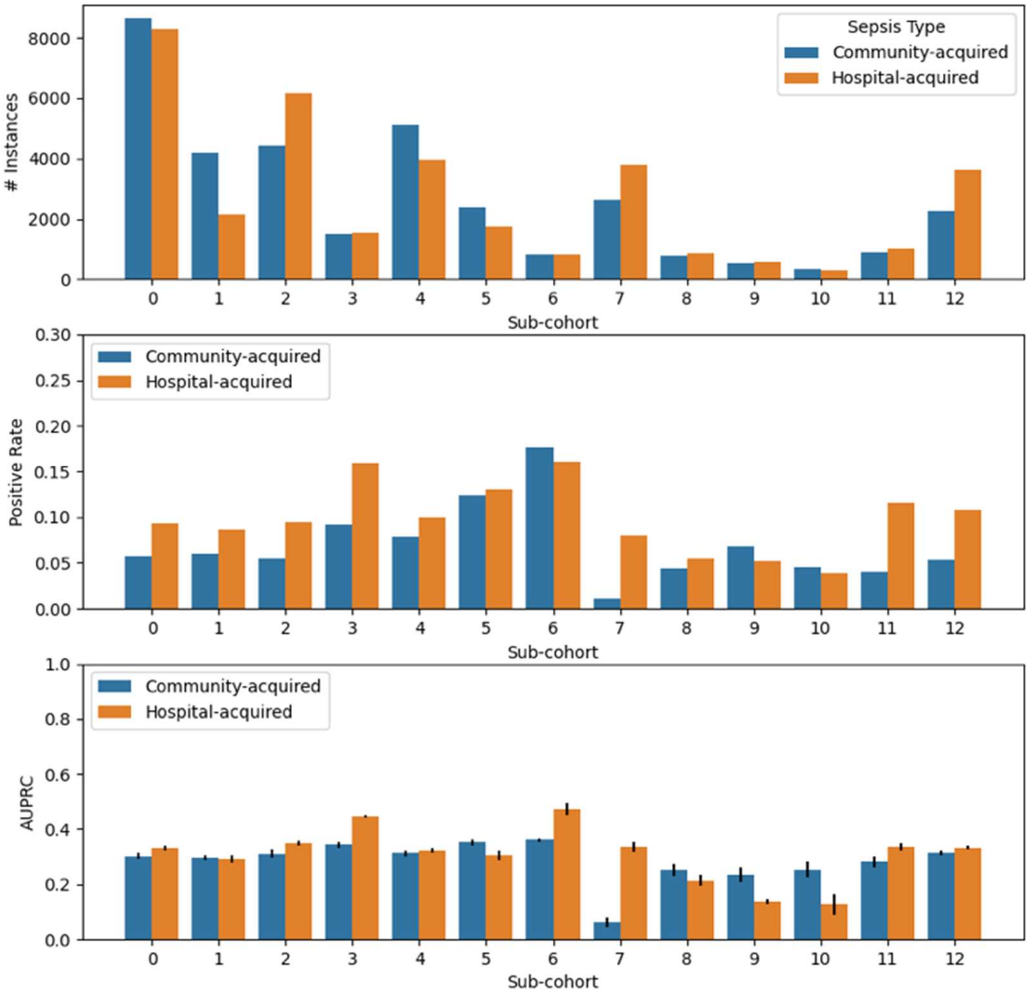

The number of sepsis episodes and the positive rates of RS and RR isolates are shown.
Definitions. SS: GNB isolates susceptible to ceftriaxone or culture negative for any GNB; RS: GNB isolates resistant to ceftriaxone and susceptible to cefepime; RR: GNB isolates resistant to both ceftriaxone and cefepime.
Subgroups.
0: Entire cohort; 1: ≥65 years; 2: <65 years; 3: History of bacterial pneumonia; 4: History of other sepsis; 5: History of disease-causing GNB such as *E. coli*, *Klebsiella* spp., and *P. aeruginosa*; 6: History of antibiotic-resistant microbes; 7: No history of 3, 4, 5, or 6; 8: Hematological malignancies; 9: Transplantation; 10: Alcoholic cirrhosis; 11: Septic shock; 12: Intubation.

© 2024 Vazquez Guillamet MC et al. *JAMA Network Open.*
