# Supplementary material for: Performance of Risk Models for Antimicrobial Resistance in Adult Patients With Sepsis
Source: JAMA Netw Open. 2024 Nov 7;7(11):e2443658. doi: 10.1001/jamanetworkopen.2024.43658 (PMC11544496; doi:10.1001/jamanetworkopen.2024.43658)
Supplement: Supplement 2. — Data Sharing Statement [file jamanetwopen-e2443658-s002.pdf]

Data Sharing Statement

Vazquez Guillamet. Performance of Risk Models for Antimicrobial Resistance in Adult Patients With Sepsis. *JAMA Netw Open*. Published November 07, 2024.
doi:10.1001/jamanetworkopen.2024.43658

Data
Data available: Yes
Data types: Data dictionary, Deidentified participant data
How to access data: https://digitalcommonsdata.wustl.edu/research-data/
When available: beginning date: 12-06-2024, end date: 12-06-2027

Supporting Documents
Document types: Statistical/analytic code
How to access documents: The analytic code is available on Github at https://github.com/HanyangLiu/sepsis-amr-jama with DOI for the repo: 10.5281/zenodo.13883230
When available: With publication

Additional Information
Who can access the data: researchers whose proposed use of the data has been approved by Washington University
Types of analyses: any purpose
Mechanisms of data availability: after approval of a proposal and with a signed data access agreement
